# Supplementary material for: Enhanced laser-driven proton acceleration via improved fast electron heating in a controlled pre-plasma
Source: Sci Rep. 2021 Jul 2;11:13728. doi: 10.1038/s41598-021-93011-3 (PMC8253820; doi:10.1038/s41598-021-93011-3)
Supplement: Supplementary file 1 — Supplementary Information. [file 41598_2021_93011_MOESM1_ESM.pdf]

Enhanced laser-driven proton acceleration via improved fast electron heating in a controlled pre-plasma (Supplemental Material)

Leonida A. Gizzi[1,2,*], Elisabetta Boella[3,4,], Luca Labate[1,2,***], Federica Baffigi[1], Pablo J. Bilbao[3], Fernando Brandi[1], Gabriele Cristoforetti[1], Alberto Fazzi[5,6], Lorenzo Fulgentini[1], Dario Giove[7], Petra Koester[1], Daniele Palla[1], and Paolo Tomassini[1]**

[1]Intense Laser Irradiation Laboratory, INO-CNR, Pisa, Italy
[2]INFN, Sez. Pisa, Italy
[3]Physics Department, Lancaster University, Bailrigg, Lancaster LA1 4YW, UK
[4]Cockcroft Institute, Sci-Tech Daresbury, Keckwick Lane, Warrington WA4 4AD, UK
[5]Dipartimento di Energia, Politecnico di Milano, Italy
[6]INFN, Sezione di Milano, Italy
[7]INFN-LASA, Segrate, Italy
[*]correspondence to leonidaantonio.gizzi@cnr.it
[**]e.boella@lancaster.ac.uk
[***]luca.labate@ino.cnr.it

ABSTRACT

Here we provide supplementary material on proton detectors and hydrodynamic numerical simulations. We also provide an extensive account of the hydrodynamic simulations of the pre-plasma induced by the pre-pulse.

Comparison of TPS and TOF detectors

Ion acceleration data were taken using the same set of ion beam diagnostics already presented in[1], including a TPS and a TOF SiC detector. The TPS and the TOF detector were used simultaneously so that a cross-comparison of the signals obtained from the two devices was possible[2,3]. In the plot of Fig.1 TPS and TOF signals are compared (see inset) for the same shot shown in Fig.2 of the manuscript for irradiation of the 25 μm thick Ti target with the pre-plasma.

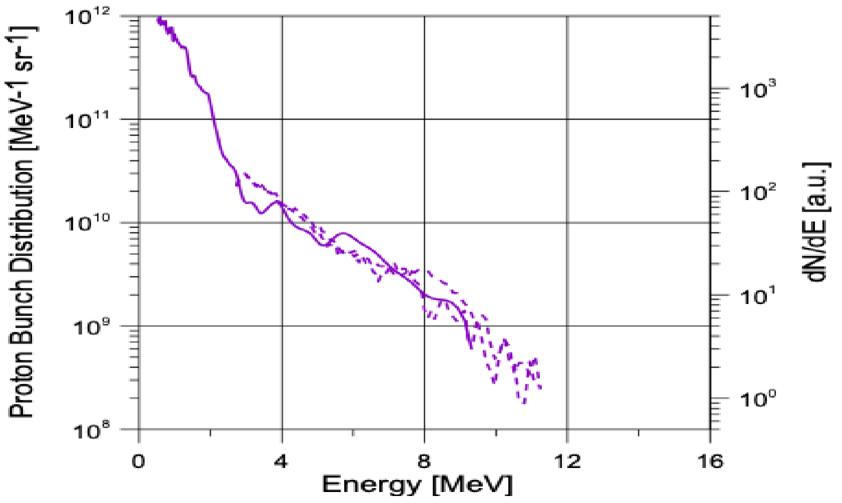

Figure 1. Experimental spectra obtained from Thomson Parabola detector (solid line) compared with the signal of the Time of Flight detector (dotted line).

According to this plot, the spectra obtained from the two detectors are in a good agreement and show the same cut-off energy of 10 MeV. We note that while the TPS is placed on the axis normal to the target, the TOF is at 1.4 deg from the target normal. So, in principle, they look at different portions of the accelerated protons. These circumstances should be taken into account when comparing the two signals.

Hydrodynamic simulations

For the modelling of the pre-pulse generated pre-plasma, we used the 2D Eulerian hydrocode POLLUX[4]. Details of the core are reported in the Methods section of the paper. Here we give details on the results concerning density profile on axis and the scale-length along the density profile.

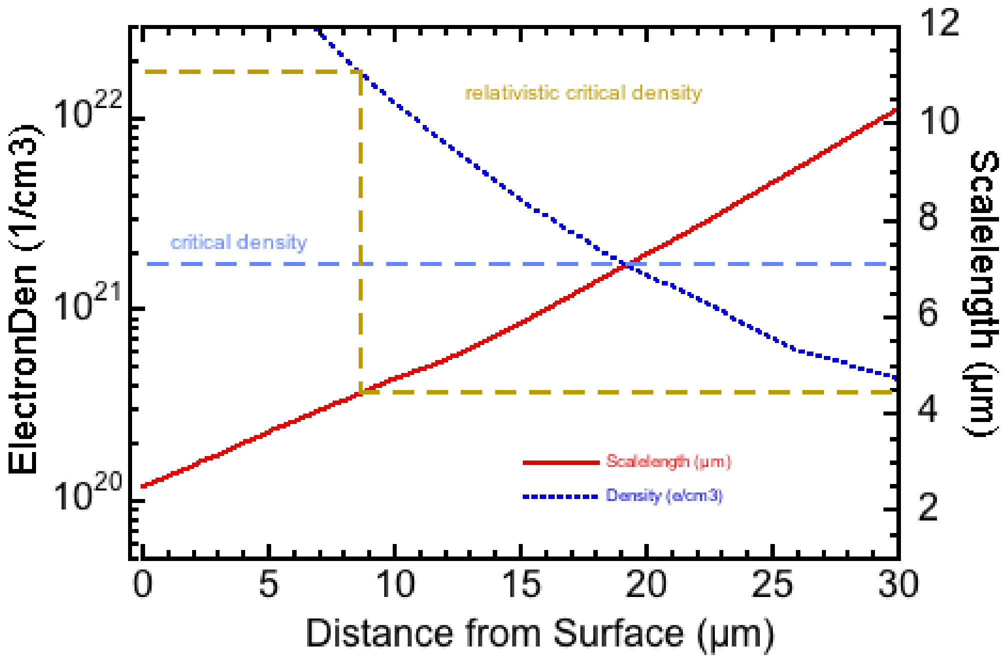

Figure 2. Electron density profile and density scale-length versus distance from the original target surface as obtained from 2D hydrodynamic simulations. A scale-length ranging from 7 to 4.5 μm is predicted between the classical (blue dashed line) and the relativistic (yellow dashed line) critical density.

A plot of both the electron density and the density scale-length at the time of arrival of the main pulse, namely after 10.4 ns, is shown in Fig.2. Here we assumed 18+ ionization of the Ti ions due to field ionization of the expanded Ti ions. Simulations predict a density scale-length of approximately 7 μm at the classical critical density and 4.5 μm at the relativistic critical density corresponding to the incident laser intensity of the main pulse with $a_0 = 10.6$.

Shock propagation into the target

The POLLUX code was also used to study the propagation of the shock created by the pre-pulse, which, once having traversed the entire target, may affect the efficiency of the TNSA process. For such purpose, simulations were carried out for a thick Ti target. Figure 3 shows the electron density maps (on a $x - r$ plane, x and r being a longitudinal and radial coordinate, respectively) at different times after the pre-pulse, as provided by the code, from 1 to 10 ns. The laser impinges from the right and the shock wave is clearly visible as the yellow crest that propagating through the solid material. Figure 4 shows the position of the shock front as a function of the time elapsed from the pre-pulse arrival, as retrieved by the POLLUX simulations. The calculated (asymptotic) value of the shock front speed is $\simeq 1.5\,\mu\mathrm{m/ns}$.

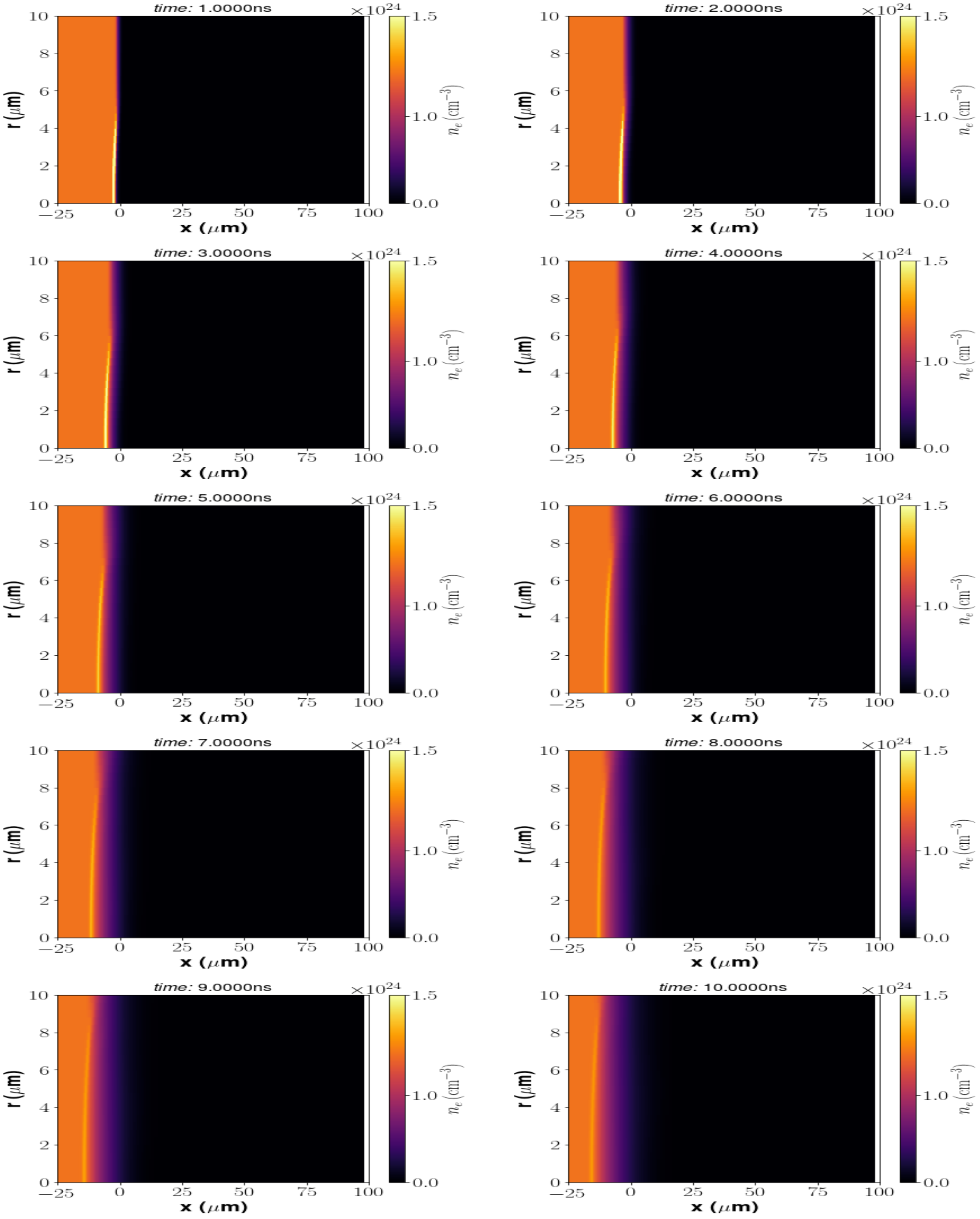

Figure 3. Maps of the electron density, as calculated by the POLLUX code, at different times, from 1 to 10 ns, after the arrival of the femtosecond pre-pulse. The *x* coordinate is along the longitudinal direction, with the pre-pulse impinging from the right. The *r* coordinate is along the radial direction. A cylindrical symmetry is assumed in the POLLUX code. The shock wave is clearly visible as a lighter feature in the target substrate.

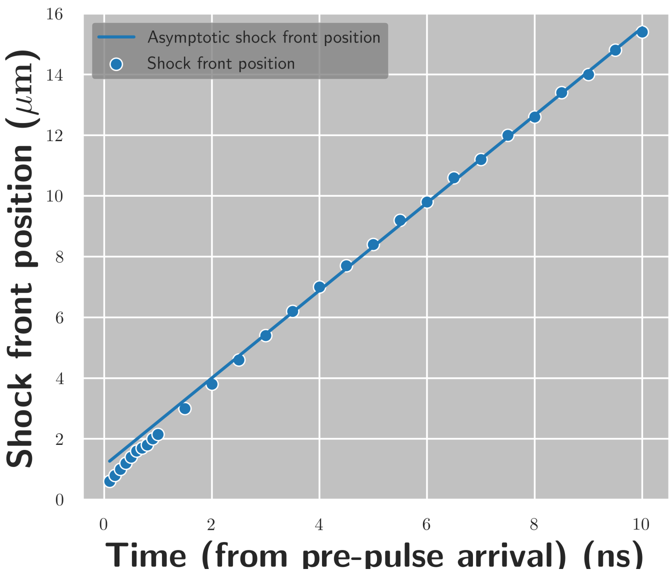

Figure 4. Shock front position as a function of time, as retrieved by POLLUX simulations of a Ti target. The 0 position corresponds to the original target surface and positive values corresponds to positions inside the (original) target.

The role of the pre-pulse timing relative to the main pulse for the two targets used in the experiment is further clarified in the diagram of Fig.5. While for the case of the 25 μm thick Ti target the main pulse impinges on target while the shock wave is still travelling inside the bulk, in the case of the thinner 10 μm Al target the main pulse arrives on target when shock break-out at the target rear face has already occurred disrupting TNSA.

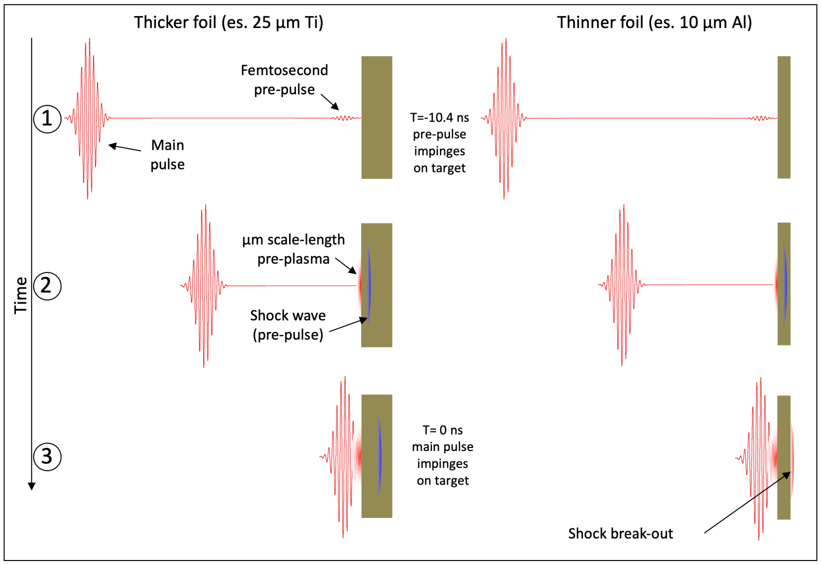

Figure 5. Diagram showing the role of the femtosecond pre-pulse and target thickness for the main pulse to pre-pulse delay used in our experiment. On the left the case of the 25 μm thick Ti target. The top-left diagram (1) refers to the time of arrival of the prepulse on target. In (2) the shock generated by the pre-pulse propagates in the target bulk. In (3) (bottom-left), the main pulse impinges on target while the shock wave is still travelling inside the bulk, On the right the case of a thinner target (e.g. 10 μm Al). The main difference is the case at time (3) (bottom-right): the main pulse arrives on target when shock break-out at the target rear face has already occurred.

References

1. Gizzi, L. A. *et al.* Light Ion Accelerating Line (L3IA): Test experiment at ILIL-PW. *Nucl. Instruments Methods Phys. Res. Sect. A Accel. Spectrometers, Detect. Assoc. Equip.* **909**, 160–163, DOI: 10.1016/j.nima.2018.03.016 (2018).

2. Gizzi, L. A. *et al.* Role of laser contrast and foil thickness in target normal sheath acceleration. *Nucl. Instruments Methods Phys. Res. Sect. A Accel. Spectrometers, Detect. Assoc. Equip.* **829**, 144–148, DOI: 10.1016/j.nima.2016.01.036 (2016).

3. Altana, C. *et al.* Investigation of ion acceleration mechanism through laser-matter interaction in femtosecond domain. *Nucl. Instruments Methods Phys. Res. Sect. A-Accelerators Spectrometers Detect. Assoc. Equip.* **829**, 159–162, DOI: 10.1016/j.nima.2016.02.016 (2016).

4. Pert, G. J. Two-dimensional hydrodynamic models of laser-produced plasmas. *J. Plasma Phys.* **41**, 263–280 (1989).
